# Analysis of Planning Strategies for Sustainable Electricity Generation in Kenya from 2015 to 2035

**DOI:** 10.1002/gch2.202100108

**Published:** 2022-05-18

**Authors:** Alex Maina, Mwenda Makathimo, George Adwek, Charles Opiyo

**Affiliations:** ^1^ Department of Energy and The Built Environment of Mount Kenya University General Kago Road Thika 342‐01000 Kenya; ^2^ School of Engineering Department of Mechanical and Industrial Engineering Masinde Muliro University of Science and Technology Kakamega 190‐50100 Kenya

**Keywords:** energy security, green house gases, LEAP, net‐present value

## Abstract

This research entails the simulation of three possible power scenarios for Kenya from 2015 to 2035 using low emissions analysis platform (LEAP). These scenarios represent the unfolding future electricity generation that will fully satisfy the demand while considering the following: energy security, power generation cost, and impacts on the environment. These scenarios are reference scenario (RS), coal scenario (CS), nuclear scenario (NS), and more renewable scenario (MRS). The findings obtained reveals that the most sustainable scenario while comparing the costs was found to be the coal scenario with a net present value (NPV) of $30 052.67 million though it has the highest greenhouse gases (GHGs) emissions. However, the more renewable scenario (MRS) has the least GHGs emissions but is found to be the most expensive scenario to implement with an NPV of $30 733.07 million.

## Graphical abstract

The paper provides in‐depth analysis of energy demand scenarios, the demand, transformation, emission, and cost analysis conducted using a low emissions analysis platform (LEAP) to determine the best possible scenario suitable for the Kenya energy space. Upon comparison of scenarios, it is realized that the coal scenario is the most favorable, the added plants size is moderate with the maximum plant capacity added being 300 MW.

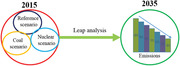

## 1 Introduction

About 1.3 billion citizens on the earth lack access to the electric grid. Of this population, about 95% live in either Africa south of the Sahara or Asia (South, Central, and East Asia).^[^ ^1^, ^2^, ^3^, ^4^ ^]^ It was estimated that 975 million people lived in Africa south of Sahara in 2014 where ≈63% lived in rural areas, and about one third of these households lacked access to the electric grid.^[^ ^5^, ^6^ ^]^ It is expected that the population will rise by 124% in Sub Saharan Africa for 20 years.

In 2015 there were 3.6 million customers connected to the national power grid in Kenya. More than 90% (3.3 million customers) were domestic, reaching more than 4 million at the end of 2015 and nearly 5 million in 2016. Use of electricity rose from 6581 to 8272 GWh from 2012/13 to 2016/17 respectively. This growth rate approximated 26% according to the updated least‐cost power development plan in Kenya for 2018.^[^ ^7^ ^]^ That has necessitated the country to review its planning strategies to keep up with the rapidly increasing population and growth domestic product (GDP) having a realization growth rate in Kenya grew with an average of 5.45% annually from 2004 until 2019.^[^ ^8^ ^]^

The installed generation capacity has increased considerably over the past five years, rising from 2298 MW in FY 2014/15 to 2712 MW in FY 2018/19, representing an annual average growth rate of 4.52%. As of December 2019, the installed capacity had increased to 2819 MW inclusive of off‐grid power. Peak demand also grew from 1512 MW recorded in FY 2014/15 to 1882 MW recorded in FY 2018/19, annual average growth of 4.89%. Peak demand of 1912 MW was recorded in October 2019. The sum of installed capacity in 2015 was 2298 MW.^[^ ^6^ ^]^ To address the challenges in the power sector in Kenya that has arisen primarily because of increased electricity demand in Kenya, major reforms have been undertaken.^[^ ^9^ ^]^ However, the lack of proper power planning raised a lot of challenges to the Kenyan economy due to the use of thermal power and thus increased green house gases (GHGs) emissions.

With this in mind, the country is dedicated to lowering the levels of GHGs emissions from the business as usual (BAU) scenario levels by 30% (143 metric tons of carbon dioxide equivalent (M_t_CO_2eq_)) by the year 2030 as envisaged by Kenya's intended nationally determined contribution, subject to being granted global financial support, trade, technological enhancement plus transfer in addition to capacity‐building.^[^ ^10^ ^]^ Therefore, the key objective of this study is to analyze the planning strategies for sustainable electricity generation in Kenya from 2015 to 2035.

## 2 Methodology

In this research, energy scenarios were modeled using a computer‐based low emissions analysis platform (LEAP).

Demand, transformation/power supply, and environmental analyses were performed over time in selected demographic, social‐economic environments to evaluate future power supply scenarios under particular policy conditions. The areas studied included; electricity supply and an analysis of the demand in Kenya in 2015, design of three scenarios, electricity sector simulation employing LEAP for the period 2015–2035, an in‐depth comparison of the alternative scenarios looking at capacity added, costs, and GHGs emissions.

### 2.1 Final Energy Demand Analysis

The determination of final energy demand was done using following categories of consumers: domestic, small commercial, medium commercial, large commercial, industrial and street lighting. A report of load forecasting was employed as a basis for the domestic load forecast.^[^ ^11^ ^]^ The level of the activity chosen for the consumer was the household as per the classification in the Kenya Power administrative regions of West Kenya, Nairobi, Coast, and Mount Kenya. The households were further classified according to location (rural and urban) and income levels (low, middle, and high).

Specific consumption for 15 clustered appliances (kWh per year) under different income and penetration levels were utilized to evaluate the domestic demand. The clustering was done from a total of 48 electrical appliances commonly in households in Kenya.

The base year demand for street lighting was obtained from the Kenya Power report.^[^ ^12^ ^]^ The base year commercial and industrial demand for the year 2015 was also obtained from the Kenya Power report.^[^ ^12^ ^]^

The elastic factor relating to the power consumption growth rate to the growth domestic product (GDP) between 2009 and 2014 was utilized to perform the projections was given by reports from Kenya economic survey^[^ ^13^ ^]^ upon which this elasticity was similarly used with the projected GDP to forecast the demand for the period from 2015 to 2035.

#### 2.1.1 Analysis for Electricity Demand and Supply for the Base Year 2015

Generation of time slices was used as the parameter for the simulation of electricity load and supply. This is a process of dividing annual electricity loads into periodic daytime divisions. The system loads were then arranged chronologically and a load duration curve (LDC) was obtained. The standard LDC was then used to develop power plant dispatch that was categorized by a merit order.

### 2.2 Scenario Design

Three scenarios developed while maintaining a reserve margin of 25%.^[^ ^14^ ^]^

These scenarios are; the coal scenario (CS), the nuclear scenario (NS), and the more renewable scenario (MRS).

### 2.3 Emission Analysis

(1) $$\begin{array}{c} {\text{Emission}}_{t}={\text{Energy consumption}}_{t,y}\times \text{Emission }\text{​}{\text{factor}}_{t,y,p} \end{array}$$

where *t* = technology type, *y* = year, and *p* = plant.

After aggregating together the emissions of every scenario were allocated a specific global warming potential relative (GWP) to CO_2_ and their effects were compared on a scenarios (Balcombe, Brandon, & Hawkes, 2018).

### 2.4 Transformation Analysis

Transmission and also distribution losses were factored in when connecting the demand side to supply. While factoring these losses the following parameters were factored in base year losses were factored as 14.5% reduced to 14% in 2016 with a reserve margin of 25% for planning.^[^ ^15^ ^]^ A load factor of 71% and constant was employed to enter the base year data.

The supply sector was modeled using various technology categories and simulations for the year 2015 were done.^[^ ^16^, ^17^ ^]^ New capacities are added in the form of exogenous capacity representing a retinue of planned additions that had a specific quantity and type. These were committed plants previously in the least cost plan that had specific time frames and endogenous capacity that represented additions of specific technologies. These were built on a need basis in order to satisfy power utilization requirements as enumerated in the demand sectors.^[^ ^18^ ^]^

(2) $$\text{Capital}\;{\mathrm{costs}}_{\text{annual}}=\text{capital}\;\mathrm{cost}\frac{i{\left(1\;\pm \;i\right)}^{n}}{\left({\left(i+i\right)}^{n}\;-\;1\right)}$$

where; *i* = discount rate; *n* = plant lifetime(in years).

Levelized annual capital costs in $ per kWh for the individual plant were calculated using Equation (3)

(3) $$\begin{array}{c} {\bar{\text{CC}}}_{t}=\frac{{\text{CC}}_{t}\times \text{CRF}}{8760\text{ hours}\times {\text{CF}}_{t}} \end{array}$$

Levelized annual fixed operation and maintenance costs in $ per kWh for individual plant were calculated as per Equation (4)

(4) $$\begin{array}{c} {\bar{\text{FC}}}_{t}=\frac{{\text{FC}}_{t}}{8760\text{ hours}\times {\text{CF}}_{t}} \end{array}$$

Total levelized annual cost in $ per kWh for the individual plant were calculated as per Equation (5)

(5) $$\begin{array}{c} \bar{{\text{AC}}_{t}}=\bar{{\text{CC}}_{t}}+\bar{{\text{FC}}_{t}}+\bar{{\text{VC}}_{t}\text{​}} \end{array}$$

Total annual costs in $ per year for the individual plant were calculated as per Equation (6)

(6) $$\begin{array}{c} {\text{TC}}_{t}={\text{AC}}_{t}\times {\text{CF}}_{t}\times 8760\times {\text{Capacity}}_{t} \end{array}$$

A comparison of costs per scenario was done and net present value (NPV) for the individual plant was calculated as per Equation (7)

(7) $$\begin{array}{c} {\text{NPV}}_{t}={\text{TC}}_{t}\frac{\left({\left(1\pm i\right)}^{n}\pm 1\right)}{i{\left(1+i\right)}^{n}} \end{array}$$

Lastly, a summation of all the plants NPV for the individual scenario was calculated as per Equation (8)

(8) $$\begin{array}{c} {\text{NPV}}_{S}=\sum_{t=1}^{n}{\text{NPV}}_{t} \end{array}$$

### 2.5 Simulation

In this process data for the demand in the following sectors, i.e., domestic, commercial, industrial, and street lighting were fed into LEAP. Equation (9) was used in the domestic sector households to calculate the aggregate demand in Kenya

(9) $$\begin{array}{c} D_{b,s,t}={\text{TA}}_{b,s,t}\times {\text{EI}}_{b,s,t} \end{array}$$

*D* = energy demand, TA = total activity (demographic data), EI = energy intensity for a specific device, *b* = branch or categories, *s* = scenario, *t* = year.

The total activity level for technology is the product of the activity levels in all branches from the technology branch back up to the original demand branch as shown in Equation 3.3.

(10) $$\begin{array}{c} {\text{TA}}_{b,s,t}=A_{b^{\prime },s,t}\times A_{b^{\prime \prime },s,t}\times A_{b^{\prime \prime \prime },s,t} \end{array}$$

*A*_b_ = activity level in a specific branch *b*, *b*′ = parent of branch *b*, *b*′′ = grandparent.

In every activity, energy intensity was calculated for every device using a criterion of the individual consumed electricity (kWh) as enumerated in the load forecast for the year 2015.^[^ ^11^ ^]^

### 2.6 Cost Analysis

Due to the constraints of the existing plants, the cost analysis a boundary for this research was restricted to the additional generation capacity for each scenario.

Annualization of capital costs over the lifetime of a plant was calculated as per Equation (11) for capital the recovery factor

(11) $$\begin{array}{c} \text{Capital}\;{\mathrm{costs}}_{\text{annual}}\;=\;\text{capital}\;\mathrm{cost}\frac{i{\left(1\;\pm \;i\right)}^{n}}{\left({\left(i+i\right)}^{n}-1\right)} \end{array}$$

*i* = discount rate, *n* = lifetime of a plant in years.

Levelized annual capital costs in $ per kWh for the individual plant was calculated as per Equation (12)

(12) $$\begin{array}{c} {\text{CC}}_{t}=\frac{{\text{CC}}_{t}\times \bar{\text{CRF}}}{8760\text{ hours}\times {\text{CF}}_{t}} \end{array}$$

Levelized annual fixed operation and maintenance costs in $ per kWh for the individual plant was calculated as per Equation (13)

(13) $$\begin{array}{c} {\bar{\text{FC}}}_{t}=\frac{{\text{FC}}_{t}}{8760\text{ hours}\times {\text{CF}}_{t}} \end{array}$$

Total levelized annual costs $ per kWh for the individual plant was calculated as per Equation (14)

(14) $$\begin{array}{c} {\text{AC}}_{t}={\text{CC}}_{t}+{\text{FC}}_{t}+{\text{VC}}_{t} \end{array}$$

Total annual costs in $ per year for the individual plant was calculated as per Equation (15)

(15) $$\begin{array}{c} {\bar{\text{TC}}}_{t}={\text{AC}}_{t}\times {\text{CF}}_{t}\times 8760\times {\text{Capacity}}_{t} \end{array}$$

To compare the costs of the scenarios, the NPV for individual plant was calculated as per Equation (16)

(16) $$\begin{array}{c} {\text{NPV}}_{t}={\text{TC}}_{t}\frac{\left({\left(1\pm i\right)}^{n}\pm 1\right)}{i{\left(1+i\right)}^{n}} \end{array}$$

Lastly, a summation of all the plants was done and the total NPV for individual scenarios calculated as per Equation (17)

(17) $$\begin{array}{c} {\text{NPV}}_{S}=\sum_{t=1}^{n}{\text{NPV}}_{t} \end{array}$$

### 2.7 Environmental Analysis

The GHGs emissions were calculated by use of emission factors while taking the values recommended by IPCC guidelines from national GHGs Inventories Tier 1.^[^ ^19^ ^]^

The technology database housed in the LEAP platform will be used in linking every technology to a corresponding amount of feedstock utilized in power generation to average the GHGs emissions.

## 3 Findings and Discussions

The findings of demand and supply analysis for year 2015 being the base year, results of the simulations, costs, GHC emissions, and the four scenarios comparisons are outlined.

### 3.1 Data Analysis for the Base Year

In 2015 the overall electricity demand was 9817 GWh. The largest user of power was the commercial and industrial sector at 55%^[^ ^12^ ^]^ as shown in **Table** **1**.

**Table 1:** Sectors power demand breakdown

Sector	Percentage consumption
Small commercial and domestic	45
Industrial and commercial	55
Street lighting	0

**Figure 1:**
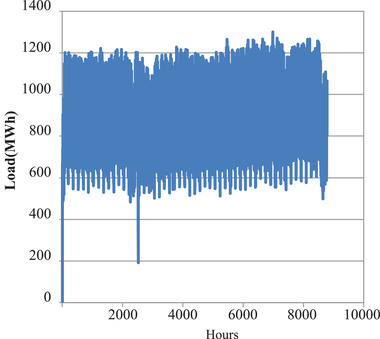
2015 Annual load duration curve.

**Figure 2:**
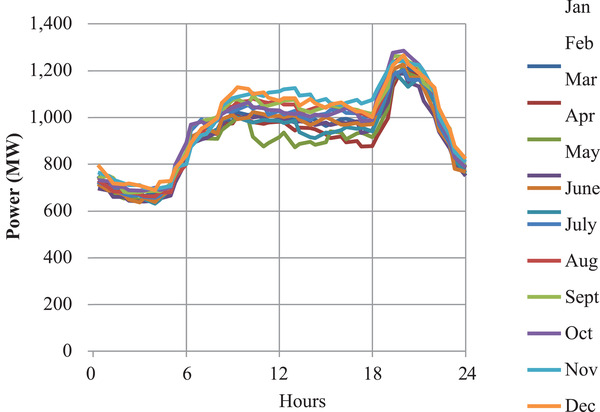
Sampled daily load duration curves.

The annual load duration curve in **Figure** **1** and the daily load duration curve in **Figure** **2** shows that the demand pattern for the year 2015 was fairly constant.

**Figure 3:**
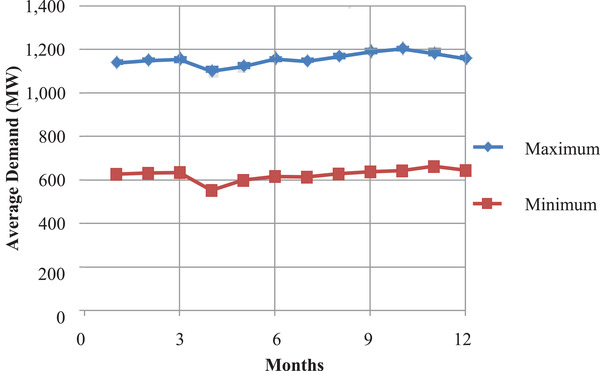
Average maximum and minimum demand.

It is also noticeable that between 1800 and 2200 h the daily peak demand occurred throughout the year. This was due to an increase in the power consumed in the domestic sector. The peak demand and the off‐peak demand were fairly constant at ≈1512 and 932 MW respectively as shown in **Figure** **3**. These findings were utilized in time slices mapping.

**Figure 4:**
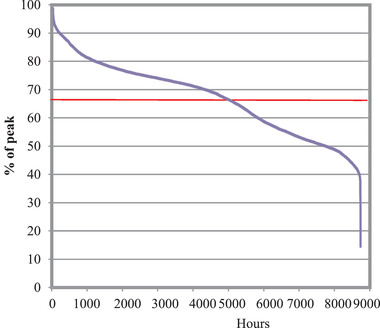
Standard load duration curve.

An annual average load factor stood at 68%. This was set as the mean height of the annual standard load duration curve shown in **Figure** **4**. This load factor helped calculate the power demand while dispatching the plants following an ascending merit order rule. This is a vital indicator of the usage of the installed capacity. Better use of the capacity was shown by a higher load factor and an idle capacity during the off‐peak hours was shown by a low load factor.

**Figure 5:**
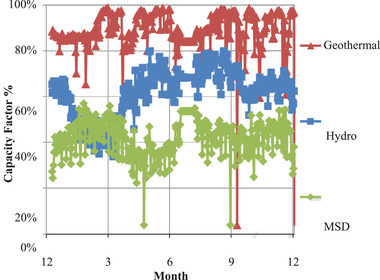
Variation of capacity factor for power plants.

On the generation side an assessment of the various power plants’ contribution to the national grid was found by determining the daily average factor for the various power categories as shown in **Figure** **5**.

The variation of the capacity factor of the power plants was used to assign the merit order for the power plants while considering the time slices. This resulted in the generation of the yearly merit order profiles. These were utilized to guide power plant dispatch.

The power production variation in a typical day is clearly shown by Figure S1 and S2 (Supporting Information) for the various classes of power plants during dry and wet seasons respectively.

It is noticeable that the production of hydro and medium speed diesel (MSD) plants varied while that of geothermal plants remained constant.

### 3.2 Simulation

For all scenarios, there was an increase in power demand from 6299 to 38 500 GWh as displayed in Figure S3 in the Supporting Information. The peak demand rose from 1286 to 7500 MW as shown in Figure S4 in the Supporting Information.

The projected growth for the RS scenario power supply is displayed in Figure S5 in the Supporting Information. At the inception stages of simulation, a capacity overdesign is noticeable but this is slowly reduced by an increase in demand. The total capacity generated in RS increases to 13 192 MW in 2035. Figure S6 (Supporting Information) displays power supply growth projection in the NS scenario. Total capacity generated increased to 14 072 MW.

Figure S7 (Supporting Information) displays the power supply growth projection in the CS scenario. The total capacity generated is 13 552 MW which is comparable to the reference scenario.

Figure S8 (Supporting Information) shows the power supply growth projection for the MRS scenario. The total capacity generated for this scenario is 15 897 MW.

Upon comparing the scenario, it was realized that the coal scenario, was the most favorable because the added plants size is moderate with the maximum capacity added being 300 MW.

While considering the nuclear scenario the added capacity, an additional 1000 MW will be realized from one nuclear plant and therefore there will be excess capacity and hence a high reserve margin. Looking at the renewable energy scenario it's actual reserve margin is optimum. However, since the reserve margin which is the basis of plant addition in this study is not realized by adding wind and solar plants, then the other plants compensate this resulting in a very large amount of capacity generated.

### 3.3 Cost Analysis

Figure S9 (Supporting Information) is an illustration of the spread of the capital costs for all the scenarios. The MRS was the highest expensive scenario while the lowest expensive scenario being CS. The large MRS scenario costs were due to the large capacity installed at 15 897 MW while comparing it to the other scenarios and also to moderately higher costs of capital of the more renewable technologies.

Figure S10 (Supporting Information) shows a comparison of the annual fixed costs. It is evident that no major variations are noticeable in the MRS, NS, and RS scenarios. The annual cost pattern showed a stable trend across the study period. A stable trend in this research was realized in the findings of the annual fixed costs pattern. The least annual fixed cost was found to be that of the CS scenario since it had an added plant capacity 300 MW and therefore the cost was spread for 30–40 years. The results showed that the CS figures were more suitable than those of the nuclear plants in the RS and NS. In the more renewable scenario, the capacity added was the highest in comparison to other scenarios and therefore high annual costs were realized. A variable costs comparison showed that the MRS had the lowest costs shown in Figure S11 in the Supporting Information. This is because that renewable energy plants portray the least variable costs because they exploit the free available natural energy resources.

While considering the discounted on all the costs relative to the base year at discounting rates of 10%, 8%, and 12% CS was found to have the lowest cost of all the discount rates followed by the RS and then NS while MRS is the most expensive scenario as displayed in Figure S12 in the Supporting Information.

In this study and while studying the cost analysis it is important to note that because the mode of power plants dispatch was the merit order and the fuel costs were not available, hence there was some undue advantage accorded to RS, NS, and CS scenarios vis‐à‐vis the MRS on the variable costs aspect of the generation of power.

Due to the unavailability of these costs for the existing plants, the merit order dispatch method was selected. While carrying out this selection, the most appropriate distich method was arrived while considering the running costs of the plants because it looks at the existing plants’ cost of fuels as well as recently added plants. Similarly, it also looks at the added fixed costs of the plants unlike the merit order method that can override costs spent by the existing plants.

### 3.4 Emission Analysis

The projections of the GHGs emissions for the scenarios are shown in Figure S13 in the Supporting Information. The unit of measure is million metric tonnes of CO_2_ equivalent. The scenario which had the highest emissions is CS considering the addition of coal and MSD plants satisfies the power demand However, the RS scenario had slightly less emissions values in comparison to the CS scenario from the beginning of year 2022.This was as a result of the introduction of a nuclear plant in the generation mix and thereby the contribution of the coal plants was hitherto reduced as the demand of power arose.

The MRS and NS scenario had very few emission values. Nonetheless, this would be realized at a cost. This is well as displayed in Table S1 in the Supporting Information. The GHG emissions reduction costs in the MRS scenario were $8.6/M_t_CO_2eq_ and those of NS scenario were $3/M_t_CO_2eq_ respectively.

Therefore, from the findings of GHGs emissions analysis devoid of policy intervention, the MRS and NS scenarios posed unattractiveness while looking at the costs vis‐a‐vis the reference scenario.

From these findings, it was observed that the most sustainable scenario that had the lowest emissions was the MRS. However, it was found to have the highest NPV of $30 733.07 million. Therefore, it is worth realizing that if the MRS scenario is implemented it would result in a GHG emissions saving of 59.3 M_t_CO_2eq_ in comparison to the RS scenario. Also since the NS is a clean technology scenario and has low emissions to, if implemented would result in a GHG reduction of 58.5 M_t_CO_2eq_ while compared to that of the RS scenario. Nevertheless, its NPV at $30 402.57 million is higher.

From the energy security point of view, the more renewable energy scenario utilizes the locally available resources only thus making it the least susceptible scenario to any external instability and price fluctuations.

While considering energy security situation and the fact that MRS scenario makes use of the locally available natural resources, it was rendered the least affected scenario to any external interference and price changes.

The coal scenario compares better since the only technology requiring fuel imports is the gas turbine plants that are also made to run on two fuels situations when there is unavailability of gas, i.e., kerosene can be used as a fuel.

The RS scenario and NS have within their mixes have nuclear and gas plants that make them have a higher susceptibility factor due to the effects of the external conditions.

From the objective of simulation other possible scenarios for Kenya while comparing them to the LCPDP in terms of energy security, costs and environmental impacts, the more renewable scenarios appeared to be the most sustainable pathways for Kenya to implement. Nonetheless, more analysis was found to be done more so on the costs as the research did not factor in the running costs of the present plants. Similarly, to enhance the environmental sustainability of the coal scenario, carbon capture, and storage technology needs to be introduced instead of the conventional coal plants used in this research.

## 4 Recommendations

This research shows that Kenya has an array of opportunities available that can be used in the expansion of power generation. However, further research is needed to ascertain the sustainable pathway that can satisfy the power demand requirements by exploiting the locally available resources at the most optimal cost.

## Conflict of Interest

The authors declare no conflict of interest.

## Supporting information

Supporting Information

## Data Availability

The data that support the findings of this study are available in the supplementary material of this article.
