# Supplementary material for: Analysis of Planning Strategies for Sustainable Electricity Generation in Kenya from 2015 to 2035
Source: Glob Chall. 2022 May 18;6(7):2100108. doi: 10.1002/gch2.202100108 (PMC9284647; doi:10.1002/gch2.202100108)
Supplement: Supplementary file 1 — Supporting Information [file GCH2-6-2100108-s001.pdf]

Supporting Information

for *Global Challenges,* DOI: 10.1002/gch2.202100108

Analysis of Planning Strategies for Sustainable Electricity Generation in Kenya from 2015 to 2035

Alex Maina, Mwenda Makathimo, George Adwek, and Charles Opiyo*

Supplementary Figures and Tables

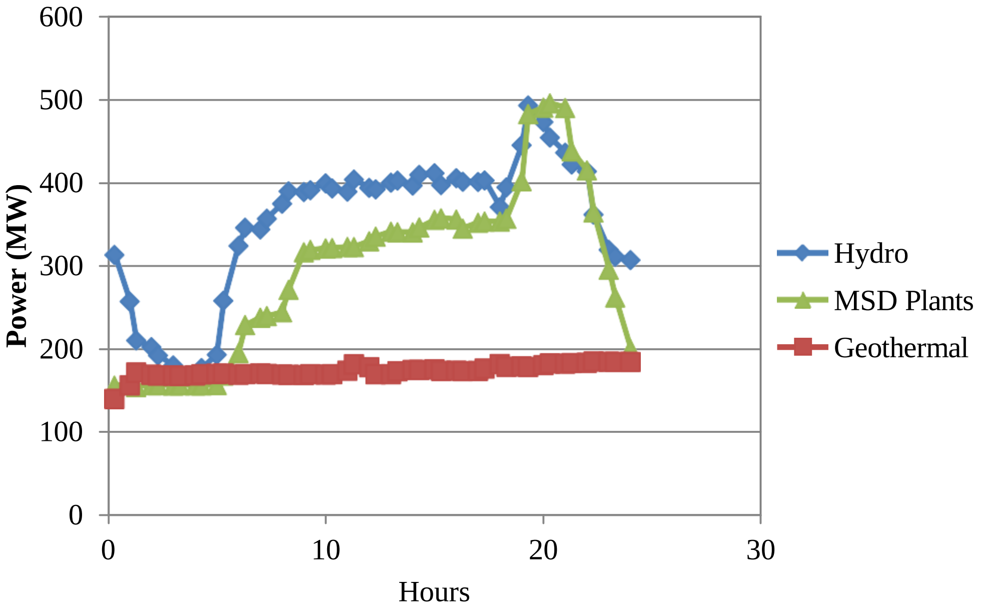

Figure S1. Typical dry season supply curve

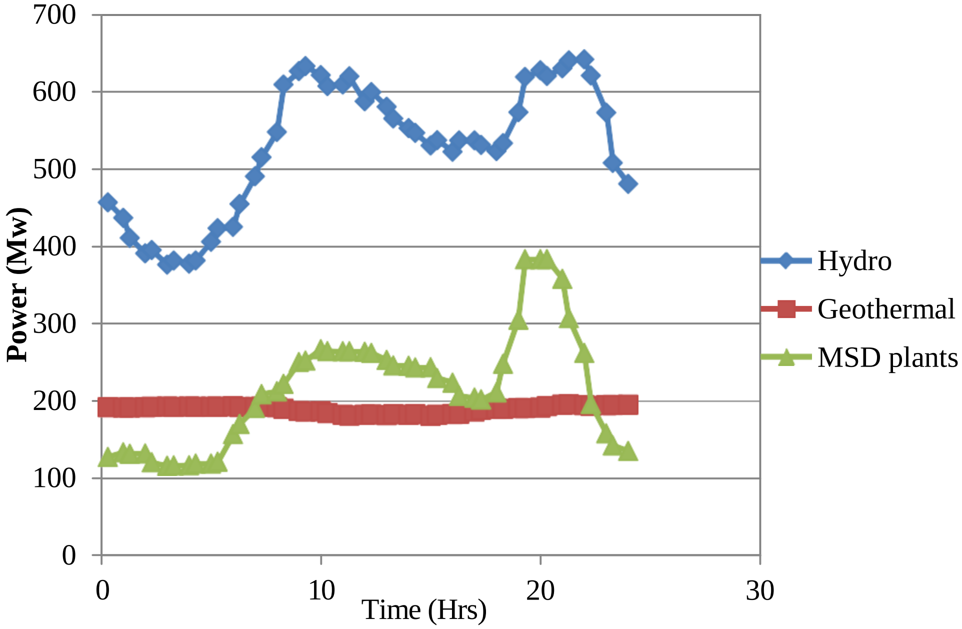

Figure S2. Typical wet season supply curve

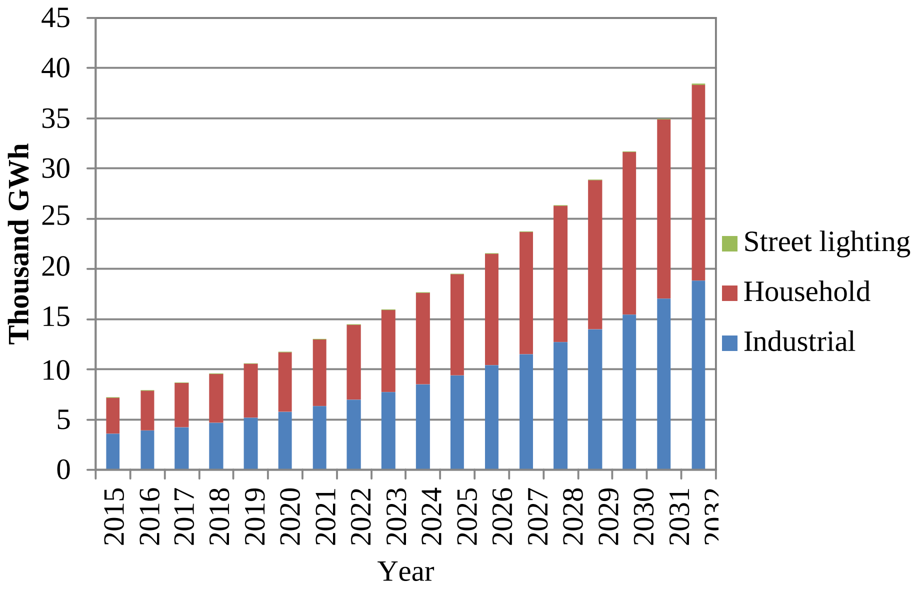

Figure S3. Electricity demand as modeled from 2015 to 2035

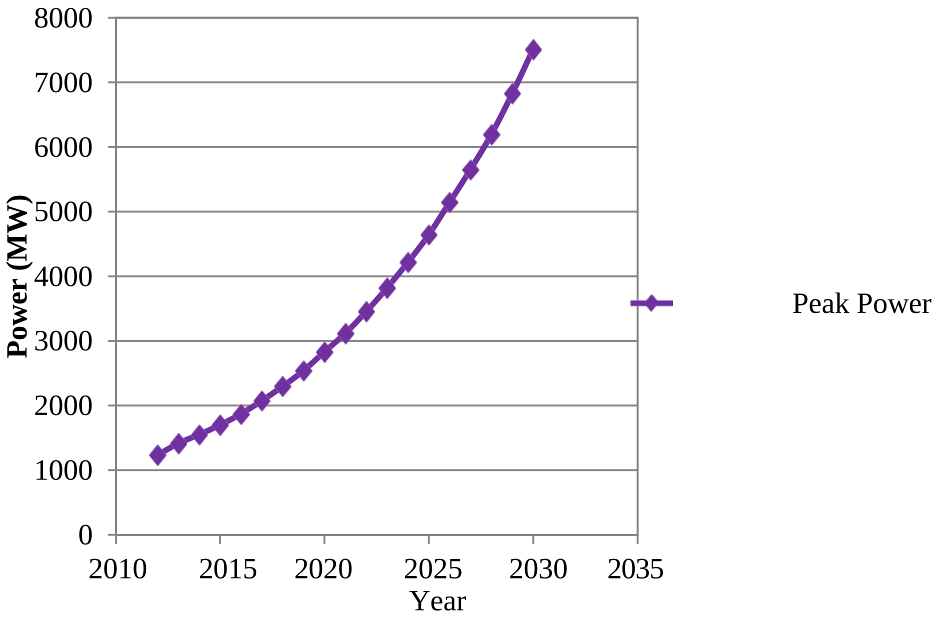

Figure S4. Projected peak power requirements from 2015 to 2035

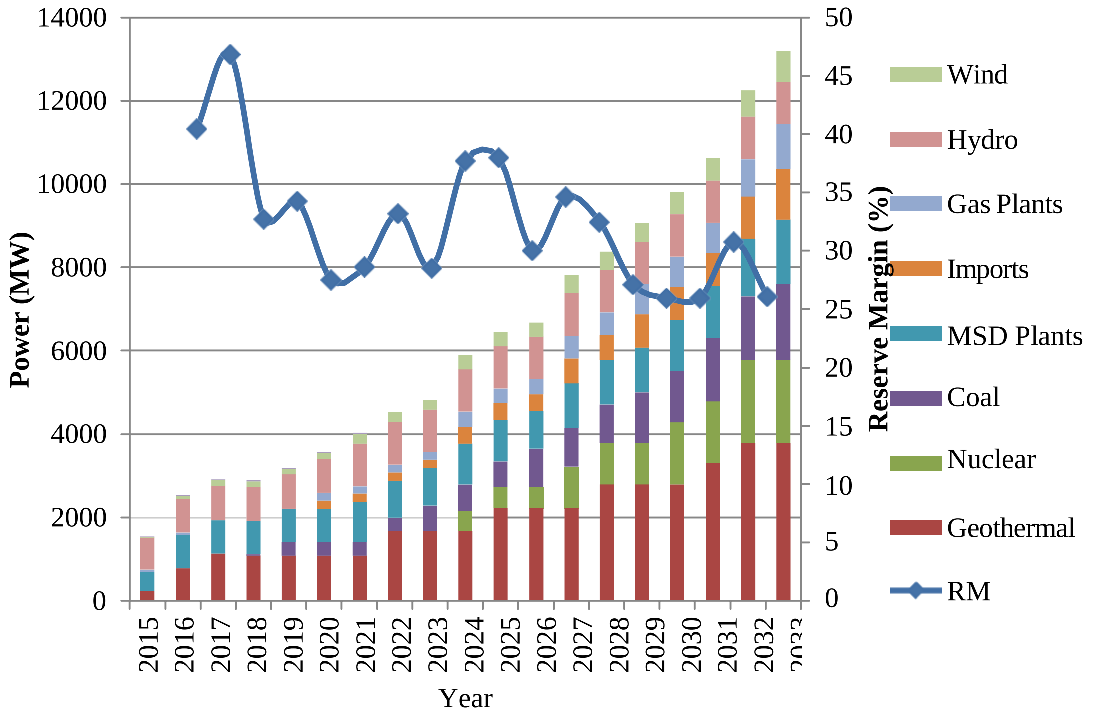

Figure S5. Projected electricity supply growth in RS scenario

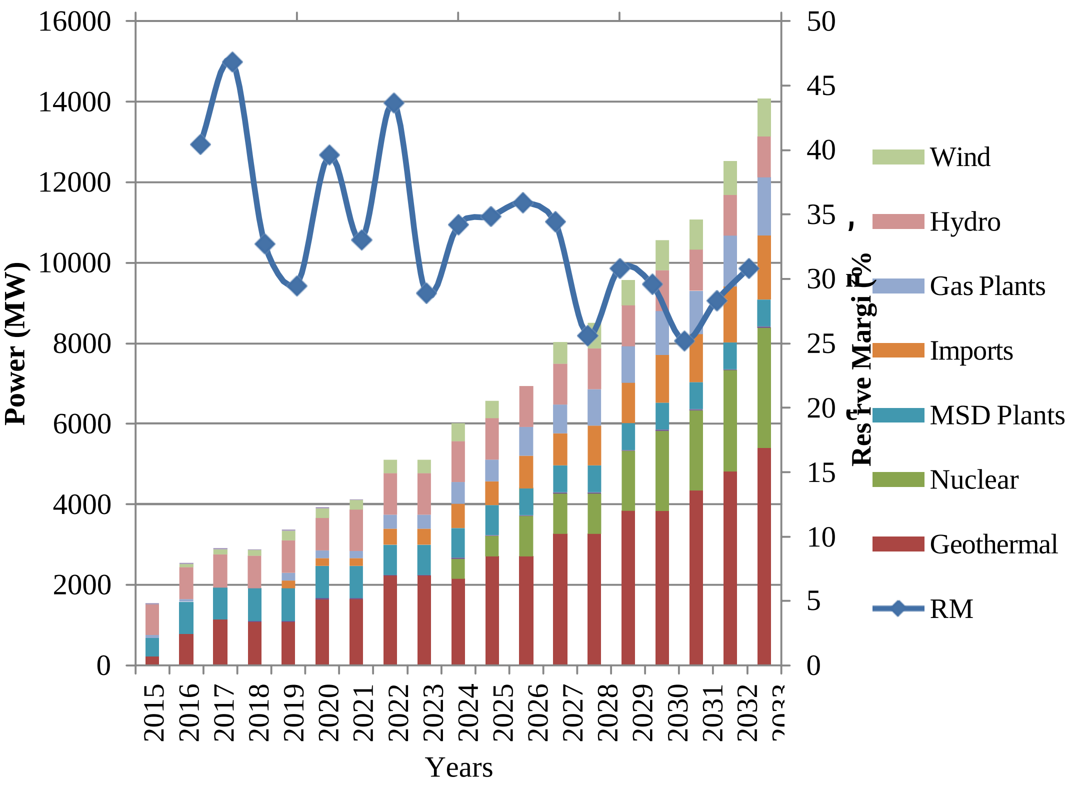

Figure S6. Projected electricity supply growth in the NS scenario

.

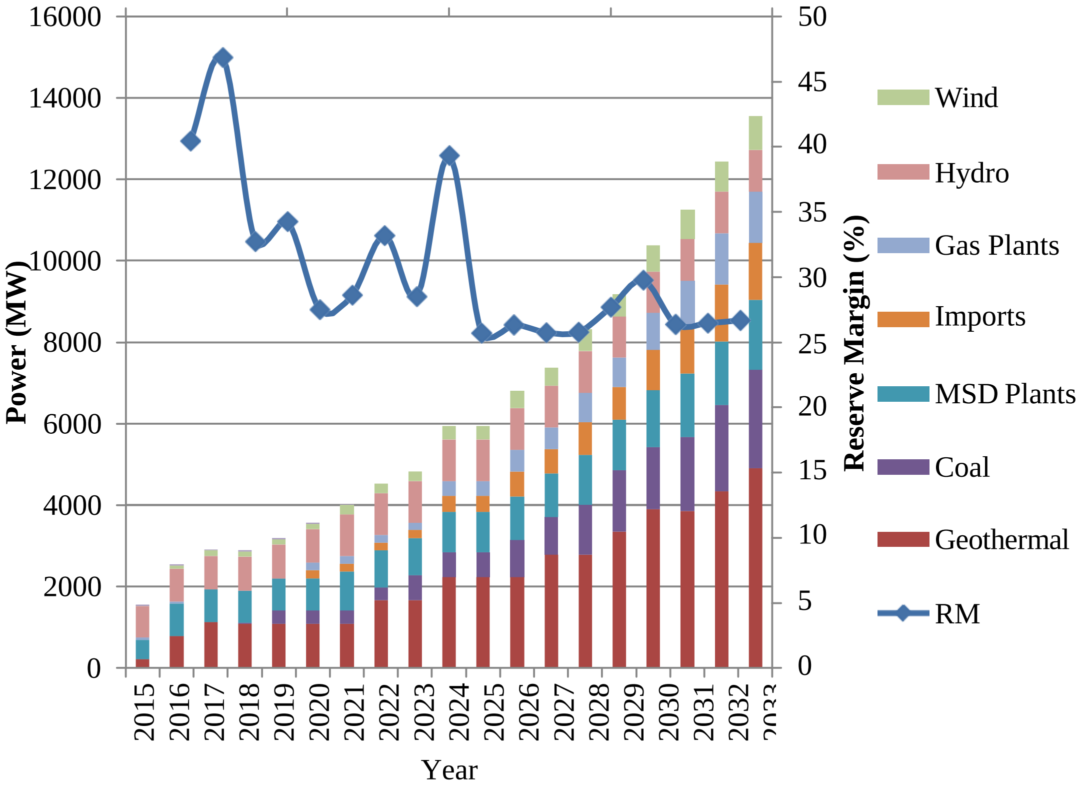

Figure S7. Projected electricity supply growth in CS scenario

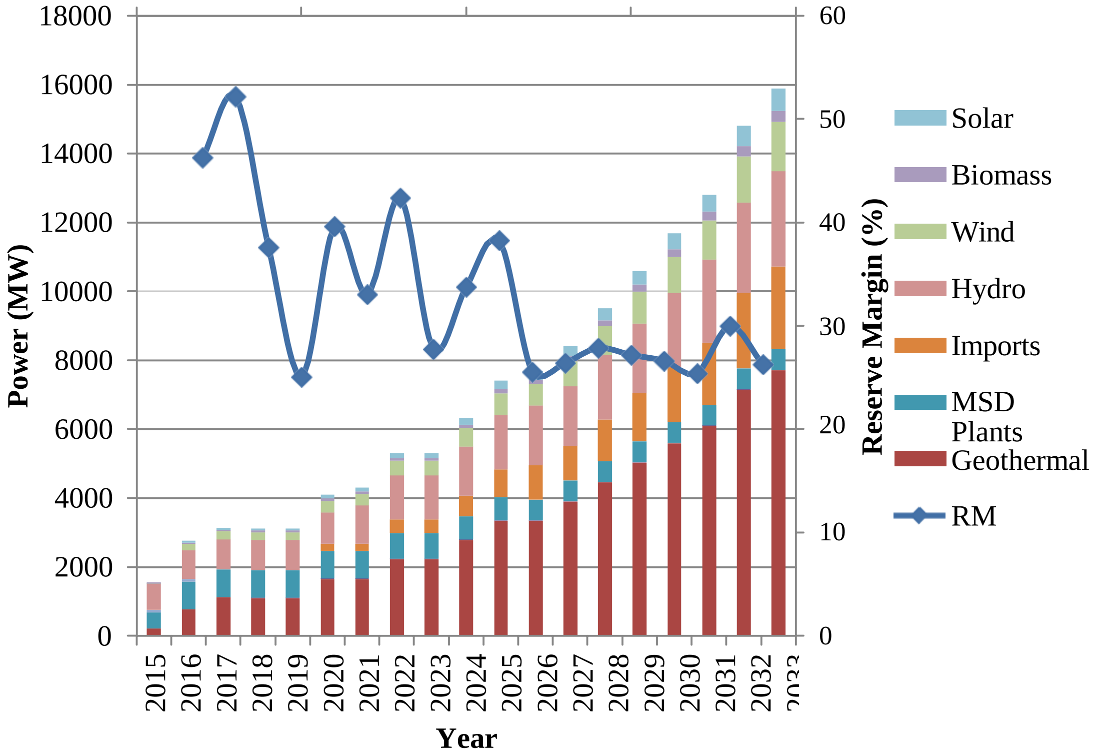

Figure S8. Projected power supply growth in the MRS scenario

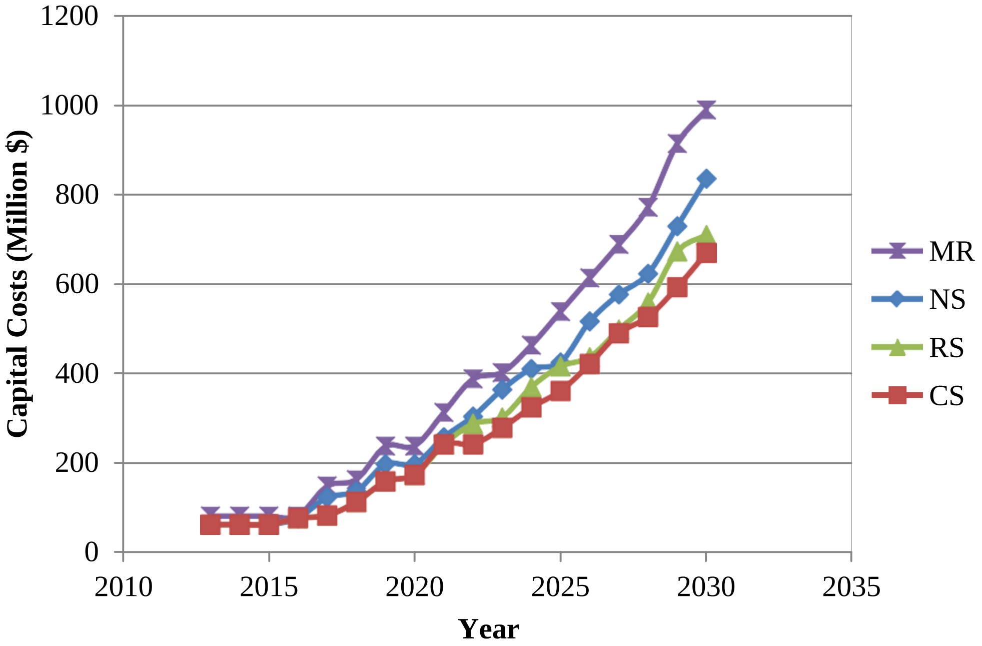

Figure S9. Capital costs comparison of the scenarios

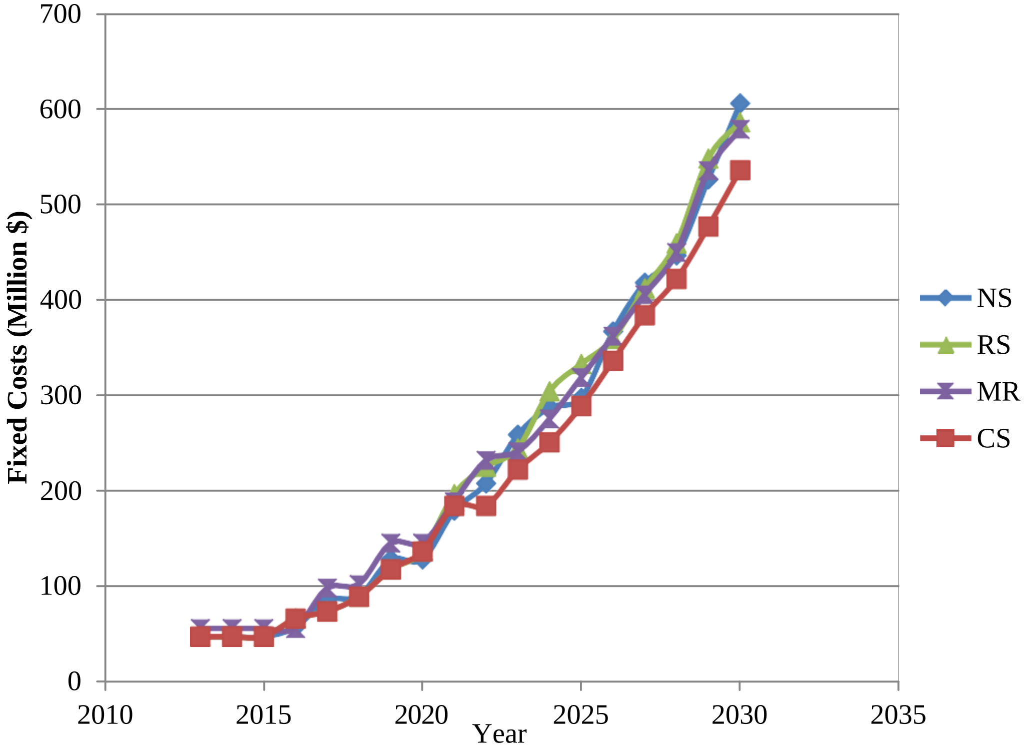

Figure S10. Fixed costs comparisons of the scenarios

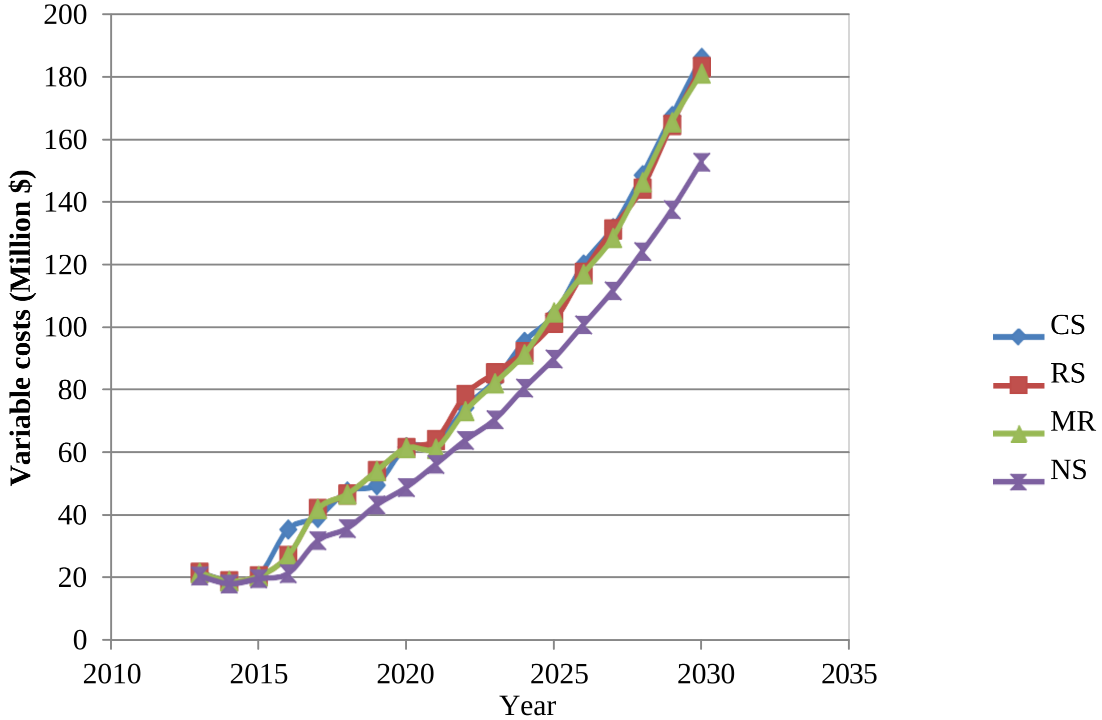

Figure S11. Variable costs comparison for the scenarios

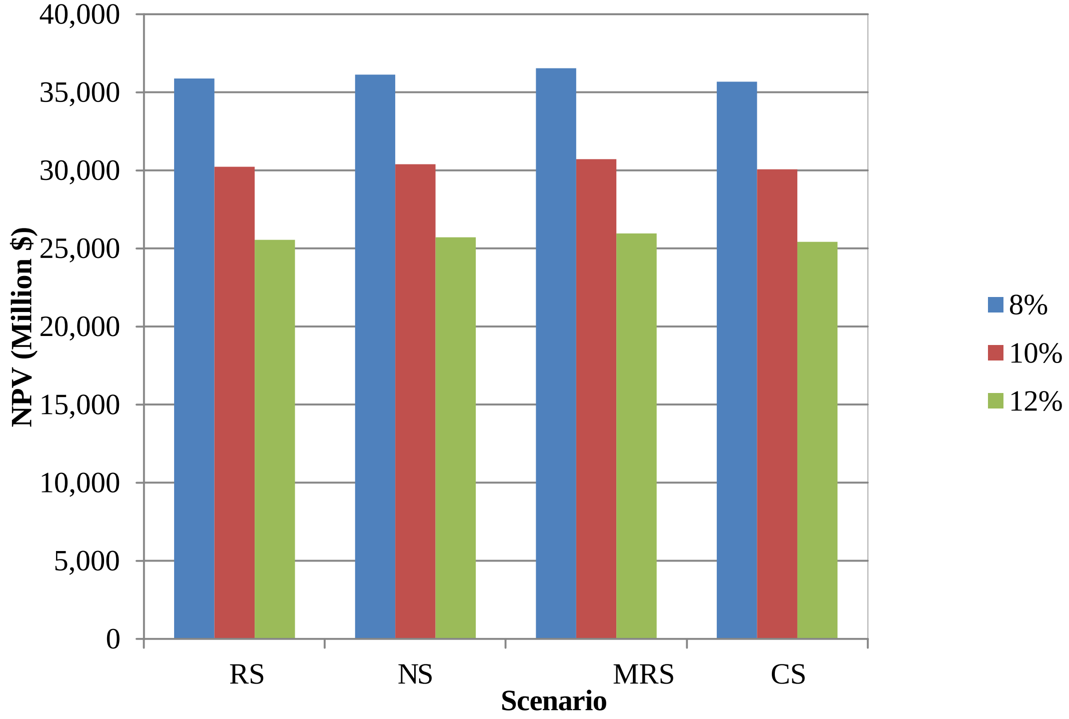

Figure S12. Net present generation costs for all scenarios

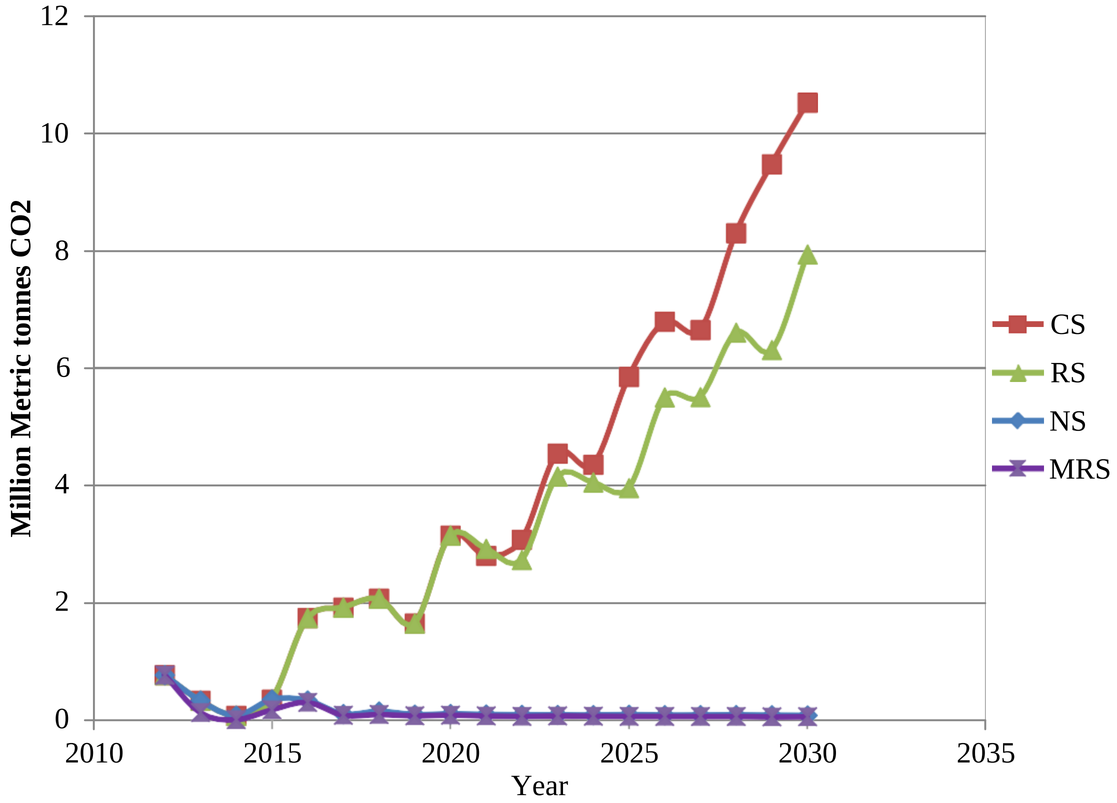

Figure S13. Projected emission levels for all scenarios

Table SM1. Cumulative cost and benefit analysis 2015 to 2035; compared to Reference Scenario

**Discount rate**	**10%**		
Scenario	MRS	NS	CS
Net Present Value (NPV) million $	507.2	176.7	-173.2
GHG Savings (Million tCO_2eq.)	59.3	58.5	-12.7
Cost of Avoided CO_2 (U.S. Dollar/tCO_2eq.)	8.6	3	n/a

Table SM2. Projected population in Kenya from 2015 to 2035

**Year**	2015	2020	2025	2030	2035
Population	46,813,114	53,460,584	60,440,476	67,812,732	75,661,869
